# Clinical improvements after treatment with a low-valine and low-fat diet in a pediatric patient with enoyl-CoA hydratase, short chain 1 (ECHS1) deficiency

**DOI:** 10.1186/s13023-022-02468-6

**Published:** 2022-09-05

**Authors:** Silvia Pata, Katherine Flores-Rojas, Angel Gil, Eduardo López-Laso, Laura Marti-Sánchez, Heydi Baide-Mairena, Belén Pérez-Dueñas, Mercedes Gil-Campos

**Affiliations:** 1 Pediatric Research and Metabolism Unit, Reina Sofia University Hospital, University of Córdoba, 14010 Córdoba, Spain; 2 Maimónides Institute for Biomedical Research of Córdoba (IMIBIC), Córdoba, Spain; 3 Department of Biochemistry and Molecular Biology II, Institute of Nutrition and Food Technology “José Mataix,” Biomedical Research Center, Parque Tecnológico de la Salud, University of Granada, Avenida del Conocimiento s/n, Armilla, 18100 Granada, Spain; 4 Instituto de Investigación Biosanitaria IBS.GRANADA, Armilla, 18100 Granada, Spain; 5 CIBEROBN (Physiopathology of Obesity and Nutrition), Instituto de Salud Carlos III (ISCIII), 28029 Madrid, Spain; 6 Pediatric Neurology Unit, Reina Sofia University Hospital, 14010 Córdoba, Spain; 7 CIBERER (Rare Diseases), Instituto de Salud Carlos III (ISCIII), 28029 Madrid, Spain; 8 Department of Clinical Biochemistry, Institut de Recerca Sant Joan de Déu, Barcelona, Spain; 9 Universitat de Barcelona, Barcelona, Spain; 10 Pediatric Neurology Research Group, Hospital Vall d’Hebrón, Barcelona, Spain; 11 Universitat Autònoma de Barcelona, Barcelona, Spain

**Keywords:** Children, Enoyl-CoA hydratase, Leigh syndrome, Diet, Valine

## Abstract

**Background:**

Enoyl-CoA hydratase short-chain 1 (ECHS1) is a key mitochondrial enzyme that is involved in valine catabolism and fatty acid beta-oxidation. Mutations in the *ECHS1* gene lead to enzymatic deficiency, resulting in the accumulation of certain intermediates from the valine catabolism pathway. This disrupts the pyruvate dehydrogenase complex and the mitochondrial respiratory chain, with consequent cellular damage. Patients present with a variable age of onset and a wide spectrum of clinical features. The Leigh syndrome phenotype is the most frequently reported form of the disease. Herein, we report a case of a male with ECHS1 deficiency who was diagnosed at 8 years of age. He presented severe dystonia, hyperlordosis, moderate to severe kyphoscoliosis, great difficulty in walking, and severe dysarthria. A valine-restricted and total fat-restricted diet was considered as a therapeutic option after the genetic diagnosis. An available formula that restricted branched-chain amino acids and especially restricted valine was used. We also restricted animal protein intake and provided a low-fat diet that was particularly low in dairy fat.

**Results:**

This protein- and fat-restricted diet was initiated with adequate tolerance and adherence. After three years, the patient noticed an improvement in dystonia, especially in walking. He currently requires minimal support to walk or stand. Therefore, he has enhanced his autonomy to go to school or establish a career for himself. His quality of life and motivation for treatment have greatly increased.

**Conclusions:**

There is still a substantial lack of knowledge about this rare disorder, especially knowledge about future effective treatments. However, early diagnosis and treatment with a valine- and fat-restricted diet, particularly dairy fat-restricted diet, appeared to limit disease progression in this patient with ECHS1 deficiency.

**Supplementary Information:**

The online version contains supplementary material available at 10.1186/s13023-022-02468-6.

## Background

Enoyl-CoA hydratase, short-chain 1 (ECHS1), EC 4.2.1.17, is also known as SCEH. It is a protein encoded by gene ID 1892, which is located on chromosome 10. The gene product is a member of the hydratase/isomerase superfamily that localizes to the mitochondrial matrix. It is broadly expressed in the liver, kidney, and adipose tissue, and its expression in 22 other tissues, including the whole gastrointestinal tract, brain, and numerous secretory glands, has been reported. Transcript variants utilizing alternative transcription initiation sites have been described [1].

**Fig. 1:**
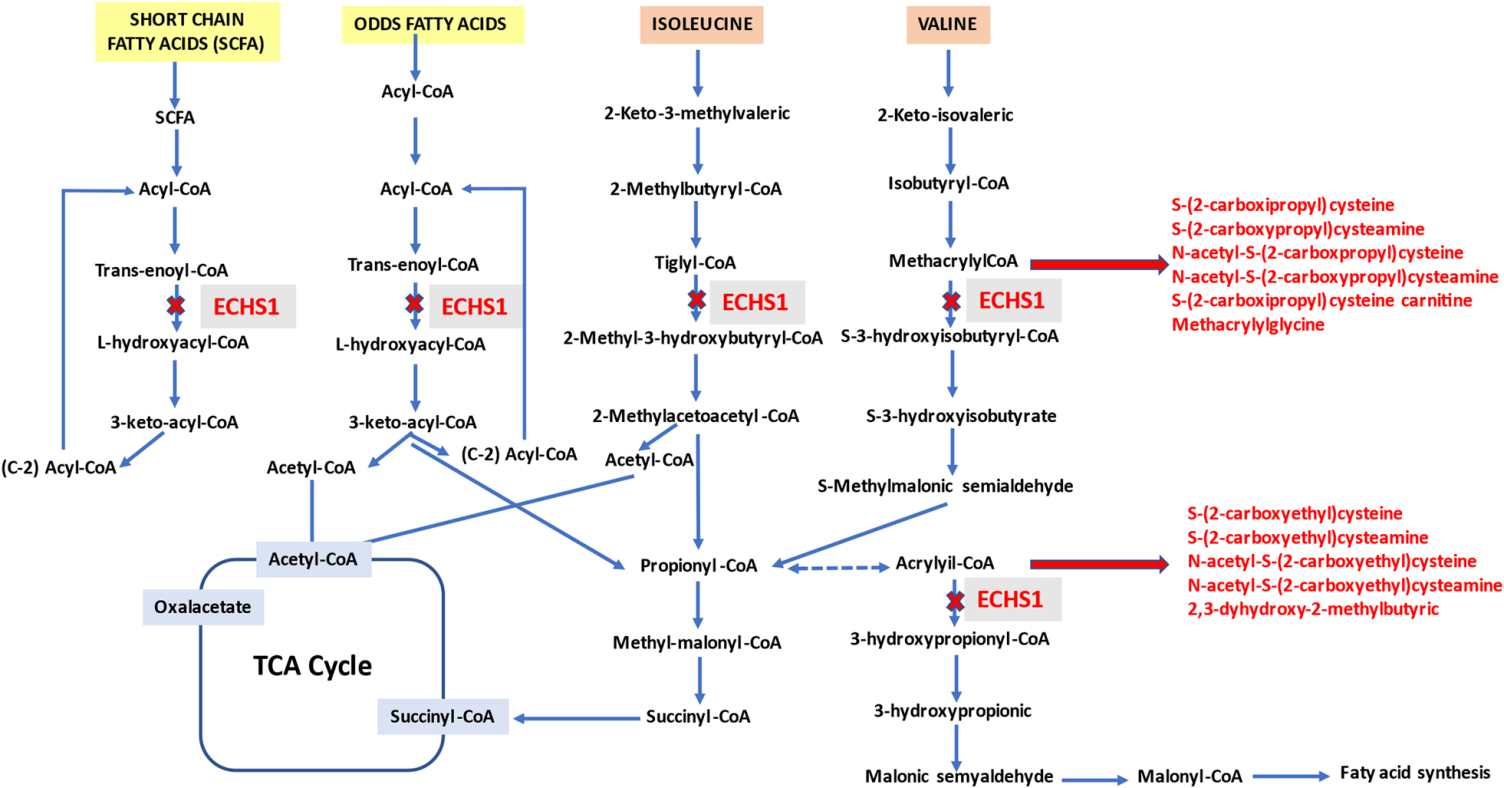
Illustration of the short-chain fatty acid, odd-chain fatty acid and valine catabolic pathways, showing the formation of certain metabolites as a result of short-chain enoyl-CoA hydratase 1 (ECHS1) deficiency. The concept that 2,3-dihydroxy-2-methylbutyric acid originates from acryloyl-CoA was indirectly inferred based on Peters et al. [6]

ECHS1 catalyzes the hydration of the double bond between the second and third carbons of 2-trans-enoyl-coenzyme A (CoA) intermediates to L-3-hydroxy-acyl-CoAs [2, 3]. It catalyzes several metabolic pathways. In particular, this crotonase catalyzes the second step of beta-oxidation of fatty acids [4] and plays a key role in valine catabolism. Furthermore, it takes part in the fourth step of this metabolic pathway by catalyzing the hydration of methacrylyl-CoA to 3-hydroxyisobutyryl-CoA and the conversion of acryloyl-CoA to 3-hydroxypropyl-CoA [5, 6]. ECHS1 is also involved in the metabolism of isoleucine in the transformation of tiglyl-CoA to 2-methyl-3-hydroxybutyryl-CoA (Fig. 1).

Mutations in the *ECHS1* gene (OMIM* 602292) result in ECHS1 deficiency, which is a rare autosomal recessive disorder that was first reported by Peters et al. [7] as a cause of Leigh syndrome (OMIM* 256000). Both the age at onset and clinical manifestations vary greatly from patient to patient. Masnada et al. [8] distinguished four phenotypes of ECHS1 deficiency based on clinical and neurological features. The first is of severe neonatal onset with a rapidly fatal course and white matter changes. The second is a severe infantile presentation with slower neurological deterioration and deep gray nuclei degeneration, developmental delay, pyramidal and extrapyramidal signs, feeding difficulties, and optic atrophy. The third phenotype is another slowly progressive juvenile form that is similar to the previous one but with less severity and mainly basal ganglia involvement. The fourth phenotype is a less common form of presentation that is based on paroxysmal exercise-induced dystonia with a normal neurological examination between these attacks and isolated degeneration of the pallidum on magnetic resonance imaging [8]. A characteristic magnetic resonance imaging (MRI) pattern of basal ganglia lesions has been reported, with small cysts in the putamen and pallidum [9]. Cardiac involvement [4] and cutis laxa are also clinical features of this disease [10].

Deficiency of ECHS1 usually leads to the accumulation of methacrylyl-CoA and acryloyl-CoA. These harmful substrates are highly reactive with sulfhydryl groups and tend to form adducts with cysteine or cysteamine [6, 7, 11]. High concentrations of these intermediate metabolites seem to cause dysfunction in the pyruvate dehydrogenase complex (PDC) and the mitochondrial respiratory chain, resulting in cellular damage [12]. Additionally, increased excretion of 2-methyl-2,3-dihydroxybutyric acid in urine is frequently observed [13]. S-(2-carboxypropyl)-cysteine, S-(2-carboxypropyl)-cysteamine and N-acetyl-S-(2-carboxypropyl)-cysteine have also been proposed as useful diagnostic markers [2, 14]. Other patients present with increased plasma levels of lactate and pyruvate in a conserved ratio [15] (Fig. 1).

Over 50 patients from more than 35 families have been reported to date [9, 16]. Whole-exome sequencing (WES) has increased the recognition of pathogenic variants in ECHS1. Most of these variants are missense variants [3], suggesting that entire loss-of-function mutations may be incompatible with life [17].

The majority of patients with ECHS1 deficiency are initially diagnosed by their clinical features and do not undergo molecular diagnosis until later in life. They usually receive standard treatments that are used for other mitochondrial diseases. Anecdotal reports of treatment with N-acetylcysteine to detoxify the reactive metabolites of methacrylyl-CoA and acryloyl-CoA have been published [18]. A valine-restricted diet is a rational biochemical approach. Recently, a valine-restricted diet appeared to be beneficial for five patients with ECHS1 defects, improving the biochemical and clinical manifestations of these disorders [19]. The administration of cofactors, i.e., coenzyme Q10, vitamins C and E, riboflavin, creatine monohydrate and other antioxidants, that are used in mitochondrial disorders with limited evidence of benefit has also been considered [18, 20, 21]. Furthermore, restricting the consumption of specific fatty acids has been tested in several FAO disorders [22]; this approach aims to reduce the synthesis of toxic intermediates [23]. In addition, although high-fat, reduced-carbohydrate ketogenic diets have been used in some cases of moderate ECHS1 deficiency, they have been ineffective in improving the symptoms in severe cases of the disease [24, 25].

Based on the previous background, here, we report the case of an ECHS1-deficient child and how the use of a valine- and fat-restricted diet partially improved his condition.

## Materials and methods

### Participant

The patient was a boy with ECHS1 deficiency who was diagnosed in 2017 in the Pediatric Neurology Unit of a third-level hospital in Spain. He was the first born from a set of twins and was delivered by scheduled cesarean section in 2006. He was conceived via the in vitro fertilization of two of the mother's eggs and the pregnancy was uneventful. His twin sister is healthy. His parents are also healthy, and their marriage is not consanguineous.

He had normal neurological development up to 15 months of age. Ten days after receiving a vaccination, he suffered from neurological regression, with acute ataxia onset, gait loss, and pyramidal signs. In addition, severe dystonia affected his daily activities, including walking, handling, and speech.

Generally, he was smiling, happy, and engaged in good social interactions. Feeding and sleep were both adequate, and the height-to-weight ratio was normal. He received speech therapy, physical therapy, and psycho-pedagogical support.

### Molecular genetic analysis

This patient was diagnosed from a multicenter project of patients with basal ganglia diseases of unknown cause [9]. The *ECHS1* variants were validated and segregated using Sanger sequencing. The genetic analysis of a whole-blood sample identified two heterozygous variants, c.123_124del p.(Gly42Glufs*3) and c.830C > T p.(Thr277Ile), in the *ECHS1* gene (NM_004092.3, sequencing). Therefore, the result was reported to be compatible with the diagnosis of Leigh syndrome.

Genomic DNA was extracted from peripheral blood samples using standard methods. The sequencing of all exons and in the flanking intronic regions ± 20 bp was performed by Centogene (Rostock, Germany) using the CentoXome Gold protocol and guaranteeing a depth of reads of 100X. Typically, 97–98% of the target bases are > 10 × covered. Next, a proprietary bioinformatics method was applied that includes annotation of bases, alignment to the hg19 reference human genome (Genome Reference Consortium GRCh37), filtering of low-quality reads and possible artifacts, and annotation of variants (Centogene, Rostock, Germany). For the medical evaluation, we considered all the pathogenic variants reported in The Human Gene Database (HGMD®) and ClinVar and all variants with minimal allelic frequency (MAF) below 1% in Genome Aggregation Database (gnomAD). All possible inheritance patterns were considered based on the clinical information contributed. Only variants related to the phenotype of the patient were reported. The patient’s clinical information and family history were used to evaluate the identified variants. All identified variants were evaluated based on their pathogenicity and causality, and those associated with the phenotype of the patient that were not benign or probably benign were reported.

Consistent with the genetic analysis, respiratory chain activity was decreased in the CII and CI + III complexes and PDC activity was decreased [9]. The results of the muscle biopsy study showed a deficit of mitochondrial respiratory chain complex I (level 8.03, pathological value < 10). Additionally, signs of mitochondrial proliferation, such as a citrate synthase value of 1353 (reference value 200–900), were observed. We only have qualitative data for PDC activity.

### Physical examination and additional tests

After the confirmation of the diagnosis and just before starting treatment, the physical examination showed the progressive development of scanning speech, severe dystonia, ataxia, hypotonia with hyperlordosis and moderate to severe kyphoscoliosis, and the extremities were in internal rotation with great difficulty walking. No abnormal eye movements were observed. The Burke-Fahn-Marsden Dystonia Rating Scale (BFMDRS) showed an 18/30 disability score.

The Gross Motor Function Classification System (GMFCS) for functional motor impairment, psychomotor delay, and intellectual disability was determined to be level IV by principal clinical judgments.

**Fig. 2:**
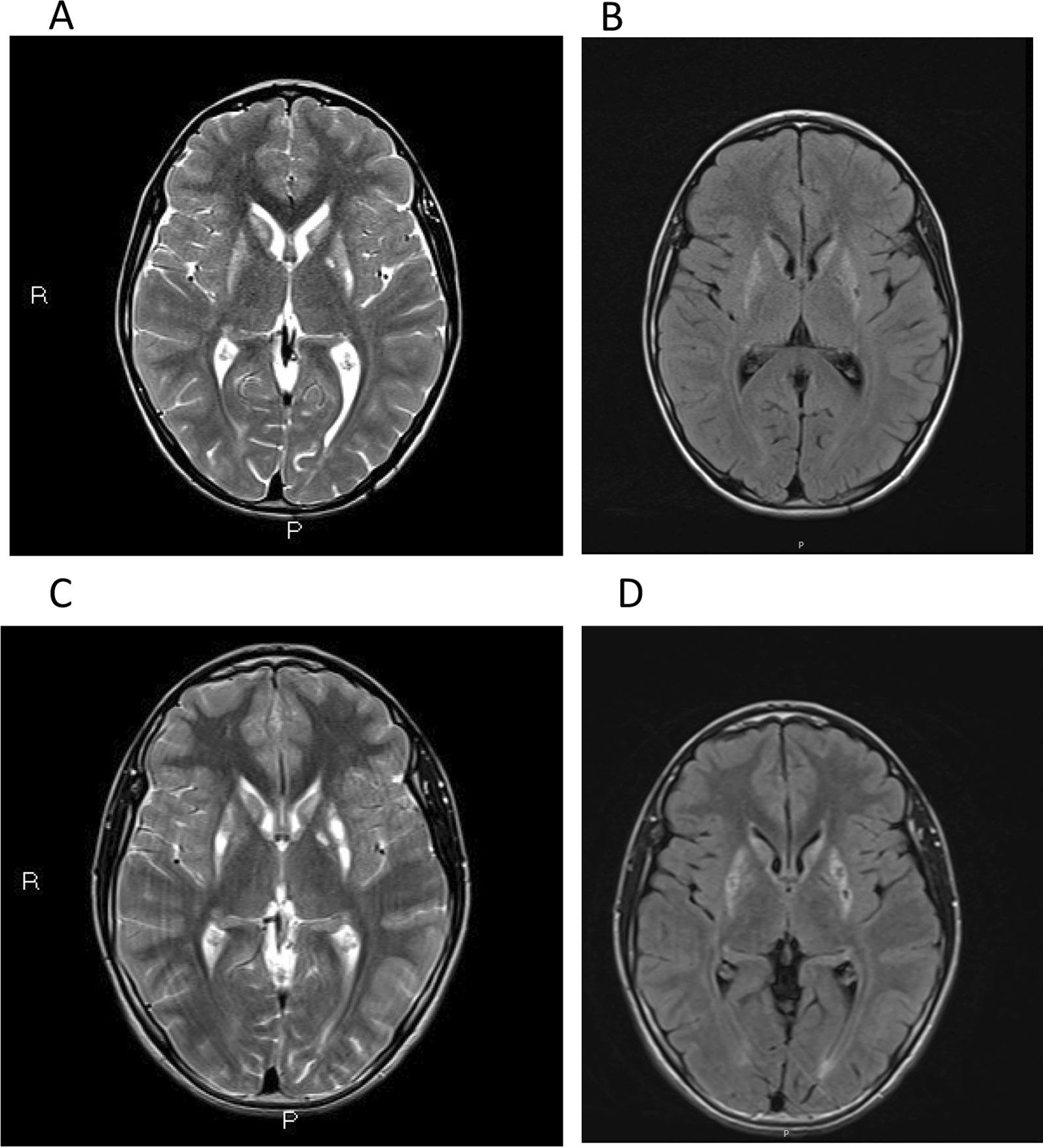
The axial T2 sequence in cerebral magnetic resonance at diagnosis (Panel **A**) and after 3 years of dietary intervention (Panel **C**) and the corresponding fluid-attenuated inversion recovery (FLAIR) images (Panel **B**, **D**)

Magnetic resonance imaging (MRI) performed in 2016 showed T2 hyperintensity in both the caudate and putamen nuclei, with more marked hyperintensity in the posterior and medial left putamen, suggesting cavitation. No brain atrophy or white matter involvement was observed (Fig. 2, Panels A and B).

Bilateral optic atrophy was detected after ophthalmic evaluation. Concerning the biochemical features, the venous acid–base analysis showed a base excess of 2.80 mmol/L, HCO_3_ 27.20 mmol/L, pH 7.44 and lactate 1.10 mmol/L. Additionally, the cerebrospinal fluid (CSF) study, including amino acid and thiamine determination, was normal, and the amino acid and acylcarnitine profiles measured in the plasma and organic acids in urine were also normal. In particular, normal urinary excretion of 2-methyl-2,3-hydroxybutyric acid was found.

### Treatment

After genetic diagnosis and based on the potentially affected metabolic pathways, the diet of this patient was modified. Valine and fat, particularly dairy fat, were restricted. This protein- and fat-restricted diet was initiated with adequate tolerance and adherence.

The nutritional plan included the consumption of a convenient, reduced volume, ready-to-drink protein substitute twice a day from 3 years of age. This substitute is also used for other congenital inborn errors of metabolism, i.e., methylmalonic and propionic acidemias. The product (MMA/PA PA cooler™) was supplied by Vitaflo®, Spain, a Nestlé Health Science company, in a 130 ml pouch (https://www.nestlehealthscience.es/vitaflo).

Table 1 shows the nutritional composition of the supplement. Indeed, the protein substitute provided all of the amino acids found in dietary protein except methionine, threonine, and valine and contained only a small amount of isoleucine. This protein substitute was used in combination with the amount of protein tolerated from the individual’s usual diet. The foods in this diet varied. However, the dairy products were from skimmed milk, and the diet was low in fatty foods. This diet also contained relatively high amounts of complex carbohydrates at each feeding, especially through the intake of bread and pasta. The natural proteins of this diet were provided by small quantities of dairy products, certain cereals, legumes (in a controlled way), gelatin, and a minimum quantity of fish. The maximum daily amount of these dietary proteins (in grams) were given to the family. In addition, free fruit and vegetables were given.

**Table 1:** Composition of the ready-to-drink protein substitute (1 pouch of 130 ml) used for the dietary management of the ECHS1 case

Energy (kcal)	103	**Minerals**	
Total amino acids (g)	18.2	Sodium (mg)	127
Protein equivalent (g)	15.0	Potassium (mg)	235
Carbohydrates (g)^1^	7.0	Chlore (mg)	182
Sugars (g)	5.9	Calcium (mg)	299
Lipids (g)	1.7	Phosphorus (mg)	267
Saturated fat (g)	0.33	Magnesium v	94
DHA (mg)	100	Iron (mg)	5.4
EPA (mg)	23	Copper (mg)	0.55
**Amino acids (g)**		Zinc (mg)	5.4
Alanine	1.68	Manganese (mg)	0.8
Arginine	1.73	Iodine (μg)	63
Aspartic acid	3.0	Molybdenum (μg)	36
Cystine	0.73	Selenium (μg)	22
Glutamine	0.00	Chromium (μg)	22
Glycine	0.79	**Vitamins**	
Histidine	1.13	A (μg)	208
Isoleucine	0.05	D (μg)	7.5
Leucine	2.37	E (mg)	3.9
Lysine	1.85	K (μg)	25
Methionine	0.00	C (mg)	27
Phenylalanine	1.13	Thiamine (B1) (mg)	0.50
Proline	0.83	Riboflavin (B2) (mg)	0.57
Serine	1.25	Niacin (mg) (B5)	6.2
Threonine	0.00	Pyridoxamine (B6) (mg)	0.70
Tryptophan	0.47	Folic acid (B9) (μg)	100
Tyrosine	1.13	B12 (μg)	1.2
Valine	0.00	Biotin (B7)	47
Carnitine (mg)	17	Pantothenic acid (B3) (mg)	2.0
Taurine (mg)	33	Choline (mg)	150

EPA, eicosapentaenoic acid; DHA, docosahexaenoic acid

^a^Sucrose, glucose syrup, and modified corn starch

A quantitative 24 h dietary record was evaluated during every visit. On average, the patient had a daily energy intake of 1312 kcal, of which 16.7% was derived from dietary protein (54.8 g/d total protein, of which 29.9 g of protein equivalent was obtained from the amino acid supplement), 70.4% (231 g/d) was derived from carbohydrates, and 12.9% (18.7 g/d) was derived from lipids.

## Results

After two years of this treatment, the child exhibited better stability. He required minimal support to walk or stand, which enhanced his autonomy at school and improved his hygiene and personal care routines. He had no adverse effects and had a good nutritional profile and growth.

After three years of treatment, his current weight is 29.5 kg (percentile 4), his height is 144 cm (percentile 10), his body mass index (BMI) is 14.04 with a prepubertal status. The family continues to perform dietary calculations as expected, complying with what was prescribed. Clinical evaluation and neurological tests are carried out every 6 months. Gross motor function has improved, and his GMFCS classification was reduced from level IV to level III. The child now shows greater stability. He requires less support when walking or standing, his language speed has increased, and he has more autonomy (i.e., he is now able to get dressed and shower alone). Minor hyperlordosis and kyphoscoliosis and fewer dystonic positions were observed, improving the ability of the patient to walk. He is even able to walk upright for 1 h a day. He turns with less difficulty, and he now has better fine motor skills and minor tremors. For example, he is now able to eat a yogurt. He has a better understanding and is conscientious of the concepts of his interest. His family seeks social integration. He is adapting to his high-school class, where he usually moves autonomously with a tricycle. On the BFMDRS, the disability score after 3 years improved from 18/30 to 9/30. His quality of life and motivation to continue this treatment have greatly increased. After treatment, there was no significant progression as evaluated by neuroimaging. In fact, MRI showed more marked hyperintensities in both the caudate and putamen nuclei, suggesting increased cavitation (Fig. 2, Panels C and D).

A video with images of the patient immediately after diagnosis and 3 years after treatment is included in the Additional file 1: Video.

Regarding the biochemical features, Table 2 shows the plasma concentrations of valine, leucine, and isoleucine at the time of diagnosis and after 1.5 and 3 years of follow-up. Valine was decreased during the clinical course compared with the baseline level, while isoleucine and leucine remained fairly constant. In the last analysis, the excretion of 2-methyl-2,3-hydroxybutyric acid was 1 nmol/mol creatinine, remaining within the normal values (< 2 nmol/mol creatinine). Additionally, no urinary methacrylyl-glycine was detected. In addition, the S-(2-carboxypropyl) cysteine concentration was 0.18 mmol/mol creatinine (normal value < 0.39) and that of S-(2-carboxyethyl)-cysteine was 0.23 mmol/mol creatinine (normal value < 0.15). However, N-acetyl-S-(2-carboxyethyl)-cysteine, N-acetyl-S-(2-carboxypropyl)-cysteine and S-(2-carboxypropyl)-cysteine carnitine were undetectable. In addition, the plasma levels of acylcarnitines were within the range of normality, and the levels of C4-OH [3-OH-butyryl/3-OH-isobutyryl-carnitine] (0.03 µmol/L) were also within the range of normality (0.01–0.06 µmol/L).

**Table 2:** The plasma concentrations of branched-chain amino acids in a case of ECHS1 deficiency treated with a valine- and fat-restricted diet

Amino acid (µmol/L)	Diagnosis	1.5 y after diagnosis	3 y after diagnosis	Range of normality
Valine	158	95	104	219 ± 47
Isoleucine	39	40	36	58 ± 15
Leucine	88	92	93	115 ± 26

## Discussion

The main finding of the present study was that treatment of male child with an ECHS1 deficiency with a valine- and fat-restricted diet resulted in an improvement in his quality of life and the amelioration of clinical features. In fact, after three years of treatment, the patient showed greater stability, required less support when walking or standing, had improved language skills, and was more autonomous.

In our patient, at the time of diagnosis, MRI detected T2 hyperintensity in the caudate and putamen nuclei. However, no brain atrophy or white matter involvement was observed. In addition, our patient exhibited bilateral optic atrophy soon after the diagnosis. In ECHS1 deficiency patients, microcephaly and impaired vision are usually detected by physical examination [9]. These patients also have difficulty fixing or following objects, nystagmus, and optic nerve atrophy, which could explain vision problems [26]. In addition, they show difficulties in speech. However, they are aware of their surroundings and communicate in other ways, such as by sounds or smiling. These patients have poor head control, so they are unable to sit up or ambulate. This symptom occurred in our patient.

Although clinical and biochemical features could suggest ECHS1 deficiency, the diagnosis of this condition is mostly established by the identification of biallelic pathogenic variants in the *ECHS1* gene by molecular genetic testing. In the present case report, the first variant in the *ECHS1* gene, c.123_124del p. (Gly42Glufs*3), alters the reading frame at codon 42. The new reading frame ends 2 positions later in a stop codon. It is classified as likely pathogenic (class 2) according to the American College of Medical Genetics and Genomics (ACMG) recommendations. The second variant in the *ECHS1* gene, c.830C > T p.(Thr277Ile), causes an amino acid change from threonine to isoleucine at position 277. In ClinVar, this variant is listed as likely pathogenic but does not provide evidence supporting its pathogenicity (clinical studies, ID: 397,546). Therefore, it is classified as a variant of uncertain significance (class 3) according to ACMG recommendations.

Urinary excretion of 2-methyl-2,3-dihydroxybutyric acid and other metabolites, such as S-(2-carboxypropyl)-cysteine, S-(2-carboxypropyl)-cysteamine, and N-acetyl-S-(2-carboxypropyl)-cysteine, is useful to diagnose ECHS1 deficiency. These compounds have been proposed as helpful biomarkers for initial screening processes [6]. Despite these observations, neither 2-methyl-2,3-dihydroxybutyric acid nor abnormal fatty acylcarnitines and organic acids in the urine were detected in our patient. Moreover, lactate levels in the plasma were not significantly elevated (1.10 mmol/L), adding evidence to the relatively mild activity of the PDC.

A definitive cure for the ECHS1 disorder has not yet been discovered. Despite the encouraging results that some studies have reported about clinical improvement in adapting patients’ diets, there is scarce literature about dietary treatment. Loupatty et al. [27] first suggested that treating patients with frequent carbohydrate-rich meals, in which the main source of energy is carbohydrates and protein intake is restricted, could be a good therapeutic option for 3-hydroxyisobutyryl-CoA hydrolase (HIBCH) deficiency. HIBCH deficiency is a mitochondrial disease that is similar to ECHS1 deficiency. Later, Soler-Alfonso et al. [28] showed that a HIBCH-deficient child clinically responded to a valine-restricted diet using a targeted formula for maple syrup urine disease. Given the clinical and pathophysiological similarities between HIBCH and ECHS1 deficiencies, it was thought that the same restricted dietary treatment could be potentially beneficial in ECHS1 subjects. According to what had been assumed, some studies demonstrated improvement in clinical outcomes after a valine-restricted diet with N-acetylcysteine supplementation [29, 30]. Recently, Abdenur et al. [19] showed enhancement in both clinical and biochemical manifestations, with a decrease in S-(2-carboxypropyl)-cysteine in patients with either HIBCH or ECHS1 deficiencies.

In the present case, dietary treatment was initiated after several tolerance tests using an available formula that was designed for patients with methylmalonic and propionic acidemia (MMA/PA). This MMA/PA cooler 15 is a ready-to-drink methionine-free, threonine-free, valine-free and low isoleucine medical food, and it was given in different amounts during the day. In addition, the patient’s diet was restricted to animal proteins, legumes, and defatted or skimmed products. However, the intake of natural proteins and carbohydrates had to be ensured. We set the consumption limit for high-biological value proteins at 1 g/kg/day. Plasma amino acids were monitored during treatment, and the family to was asked to adjust the diet when low levels were detected.

In our case, the aim of specifically restricting valine was focused on reducing neurotoxicity due to enzymatic blockade and the accumulation of metabolites in the catabolic pathway.

Mitochondria play a key role in the consumption of energy in eukaryotic cells by oxidizing fatty acids and sugars to generate ATP. Mitochondrial fatty acid β-oxidation (FAO) and oxidative phosphorylation (OXPHOS) are two key pathways involved in this process, and ECHS1 is a key enzyme involved in mitochondrial FAO. For the very-long-chain and long-chain fatty acid (C14–24) fatty acyl-CoA esters, the second, third and fourth steps of β-FAO are performed by the multidomain mitochondrial trifunctional protein (MTP). However, medium-(C8–C12) and short-chain (C4–C6) fatty acyl-CoA esters are metabolized via a different set of enzymes that perform hydration (step 2; ECHS1).

Disruption of FAO can cause human disease. However, patients with deficiencies in the FAO enzyme ECHS1 are typically diagnosed with Leigh syndrome, which is normally associated with OXPHOS dysfunction [31]. In our patient, the result of the muscle biopsy study showed a deficit of the mitochondrial respiratory chain complex I (level 8.03, pathological value < 10) together with signs of mitochondrial proliferation, such as a citrate synthase value of 1352.98 (reference value 200–900). For PDC activity, we only have qualitative data that supports mild activity. Interestingly, there may be a correlation between the ECHS1 phenotypic severity and PDC activity [31]. Patients with low PDC activity also have high lactate levels. Several of these patients present with a more severe prognosis [4, 7, 32], including death within 48 h of birth [2, 33]. In contrast, milder cases of ECHS1 deficiency do not show reduced PDC activity or lactic acidosis [6, 12, 34, 35].

Since ECHS1 deficiency is associated with severe acidosis and low ATP from impaired aerobic oxidation usually occurs, we hypothesized that a diet with a relatively low content of fat and a particularly low content of medium- and short-chain fatty acids might be beneficial for patients with impaired OXPHOS.

Restricting the consumption of specific fatty acids has been tested in several FAO disorders [22], aiming to reduce the synthesis of toxic intermediates [23]. In addition, although high-fat, reduced-carbohydrate ketogenic diets have been used in some cases of moderate ECHS1 deficiency, they have been ineffective in improving the symptoms of the disease in severe cases [24, 33].

Bovine milk lipids contain approximately 12 fatty acids in amounts greater than 1%. Butyric acid (4:0) and caproic acid (6:0) are the most important substrates for ECHS1, and they represent 11.8% and 4.6%, respectively, of the total triacylglycerol fatty acids. In addition, all of the 4:0, 83% of 6:0 and 63% of 8:0 are esterified at the sn-3 position and are easily digested by gastric and pancreatic lipases [36, 37]. These short and medium-chain fatty acids reach the systemic circulation and freely diffuse across the plasma membrane. They enter directly into mitochondria without the assistance of the carnitine shuttle system [38]. They are rapidly oxidized in the liver, muscle, and other peripheral tissues. Hence, reducing the amount of total fat, particularly dairy fat, in the diet could reduce the impact of FAO on the symptoms of mitochondrial ECHS1 deficiency.

A limitation of this study is that important biochemical markers, such as S-(2-carboxypropyl)-cysteine, S-(2-carboxyethyl)-cysteine, N-acetyl-S-(2-carboxyethyl)-cysteine, N-acetyl-S-(2-carboxypropil)-cysteine, and S-(carboxypropyl)-cysteine carnitine, were not measured at the beginning of dietary treatment, so we cannot compare them with current levels. Another limitation of our study is that the patient has a mild phenotype, as evidenced by low levels of altered metabolites as well as an improved clinical course. In only two years of treatment, the patient has progressed from great difficulty walking, severe dystonia and hypotonia with severe hyperlordosis and kyphoscoliosis to showing better stability, less dystonic positions, and much more autonomy in walking and daily routine. He has also demonstrated great social integration in school due to more fluent language and better understanding.

In this rare disease, clinical results should be considered with caution because there is no literature about how to perform a clinical follow-up and what tests could be performed to best carry out a specific evaluation to control its progression or improvement. The validated international tests used, BFMDRS and GMFCS, were applied to perform a neurological evaluation based on motor function and disability. The BFMDRS gives a disability score, and it is a universally applied instrument for the quantitative assessment of dystonia in both children and adults. It should be used longitudinally, as it was used for this patient, every 6 months, and the age of the patient should be considered. The GMFCS measures the changes in gross motor function in children with cerebral palsy or similar symptoms. Therefore, it is used both to describe a child’s current abilities and to quantify changes in function over time as a result of development, therapy, or training.

In addition, extensive biochemical evaluation, particularly those that are related to the metabolites associated with ECHS1 deficiency, was not performed by clinicians before treatment. This is because of difficulties in the hospital in carrying out specific biochemical analyses but also because of scarce knowledge about the biochemical pathways related to this ultrarare disease. Moreover, the direct effect of a low-valine and low-fat diet on those metabolites in the brain cannot be directly known, and perhaps these metabolites are found in different concentrations in the brain than in blood. Therefore, whether the diet resulted in biochemical amelioration, as it seems to have done to an extent, cannot be proven in the present case.

Indeed, the clinical improvements after 3 years on this low-valine, low-fat diet could be a fortuitous result, as we did not obtain clear biochemical evidence of improvement. However, the progression of the patient, prior to the genetic diagnosis, was evident, and clinical stabilization with some improvements would seem to be associated with our reasonable assumption of a direct effect of the diet. In the future, we hope to be able to provide new evidence related to the continuity of the diet in the long term since it does not cause adverse effects. Through these future studies, its effect can be confirmed.

## Conclusion

There is still a substantial lack of knowledge about this rare disorder. especially knowledge of future effective treatments. The dietary approach of ECHS1 has shown benefits in multiple patients. However, the correct formulation between a valine-restricted diet or a combined valine-fat restriction will need additional patient information. Indeed, early diagnosis and the therapeutic use of a valine- and fat-restricted diet supported by a specific and longitudinal clinical evaluation and metabolite measurements could help to confirm its effects on clinical disease progression in patients with ECHS1 deficiency.

## Supplementary Information

**Additional file 1**. This video shows the clinical evolution of the patient with ECHS1 deficiency before and 3 years after treatment with a low-valine and low-fat diet. One can observe how after treatment he shows better stability, less walking and turning difficulties, an improvement in fine motor skills and tremors.

## Data Availability

All data generated or analyzed during this study are included in this published article and its Additional files.

## Abbreviations

ACMG: American College of Medical Genetics and Genomics
BFMDRS: Burke-Fahn-Marsden Dystonia Rating Scale
BMI: Body Mass Index
CFS: Cerebrospinal fluid
CoA: Coenzyme A
C4OH: 3-OH-isobutyryl carnitine
ECHS1: Enoyl-CoA hydratase, short-chain 1
FAO: Fatty acid oxidation
GMFCS: Gross motor function classification system
GnomAD: Genome aggregation database
HGMD®: The human gene database
HIBCH: 3-Hydroxyisobutyryl-CoA hydrolase
MAF: Minimal allelic frequency
MMA/PA: Methylmalonic and propionic acidemia
MRI: Magnetic resonance imaging
MS: Mass spectroscopy
MTP: Mitochondrial trifunctional protein
OXPHOS: Oxidative phosphorylation
WES: Whole exome sequencing
